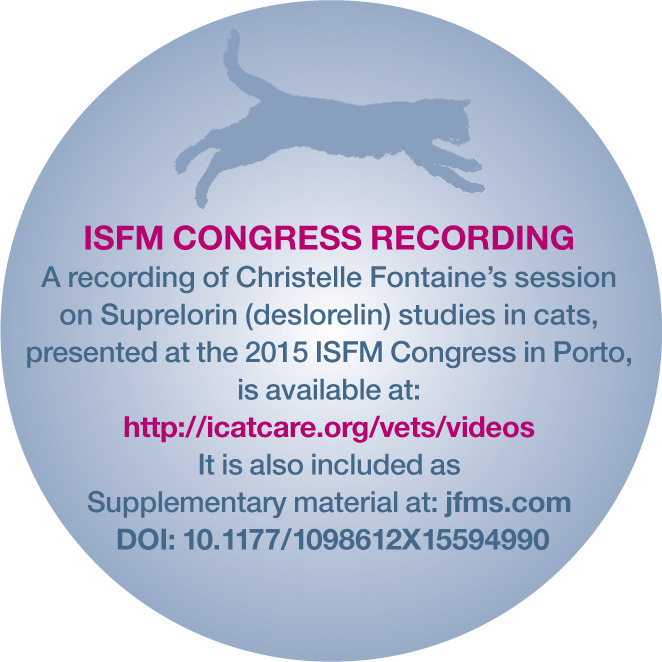# Long-term contraception in a small implant: A review of Suprelorin (deslorelin) studies in cats

**DOI:** 10.1177/1098612X15594990

**Published:** 2015-08-25

**Authors:** Christelle Fontaine

**Affiliations:** Medical Manager – Companion Animal Medical Department, Virbac, France

## Abstract

**Rationale:**

Deslorelin (Suprelorin®; Virbac) is a gonadotropin-releasing hormone (GnRH) agonist licensed in select countries for the long-term suppression of fertility in adult male dogs and male ferrets. This article summarizes studies investigating the use of deslorelin implants for the long-term suppression of fertility in male and female domestic cats.

**Evidence base:**

Slow-release deslorelin implants have been shown to generate effective, safe and reversible long-term contraception in male and female cats. In pubertal cats, a 4.7 mg deslorelin implant suppressed steroid sex hormones for an average of approximately 20 months (range 15–25 months) in males and an average of approximately 24 months (range 16–37 months) in females. Reversibility has been demonstrated by fertile matings approximately 2 years post-treatment in both male and female adult cats. In prepubertal female cats of approximately 4 months of age, puberty was postponed to an average of approximately 10 months of age (range 6–15 months) by a 4.7 mg deslorelin implant.

**Challenges:**

The large variability in the duration of suppression of gonadal activity makes the definition of the optimal time for reimplantation quite challenging. In addition, the temporary stimulation phase occurring in the weeks following deslorelin implantation can induce in adult female cats a fertile estrus that needs to be managed to avoid unwanted pregnancy. Longer duration and larger scale controlled field studies implementing blinding, a negative control group and a carefully controlled randomization to each group are needed. Furthermore, the effects of repeated treatment need to be investigated. Finally, the effect of treatment on growth and bone quality of prepubertal cats needs to be assessed. However, the ease of use, long-lasting effects and reversibility of deslorelin implants are strong positive points supporting their use for controlling feline reproduction.

## Introduction to deslorelin

Deslorelin (the active ingredient in Suprelorin^®^ 4.7 mg and Suprelorin^®^ 9.4 mg; Virbac) is a synthetic gonadotropin-releasing hormone (GnRH) agonist that is seven times more potent than GnRH.^1^ Prolonged stimulation of GnRH receptors by deslorelin leads to desensitization of these receptors.^2^ This results in a lack of synthesis and/or lack of release of the gonadotropins luteinizing hormone (LH) and follicle-stimulating hormone (FSH), inducing temporary infertility in treated individuals.^3^

The 4.7 mg deslorelin implant is registered under the brand name Suprelorin in the European Union (EU), Australia and New Zealand for the long-term suppression of fertility in adult male dogs. The EU and Australia have further registered the 9.4 mg implant for chemical castration of adult male dogs and adult male ferrets; Australia has additionally approved the implant to manage hyperadrenocorticism in ferrets. (Suprelorin^®^ F, a 4.7 mg deslorelin implant, is available in the United States as a Food and Drug Administration Indexed Product to manage adrenal disease in sterilized and sexually intact male and female ferrets.) Suprelorin 9.4 mg contains a double dose of deslorelin and a matrix without the excipient sodium acetate anhydrous to allow for a longer duration of infertility.

As the GnRH amino acid sequence is highly conserved and as desensitization always occurs following continuous exposure to GnRH agonists (such as deslorelin), several research teams have investigated the potential of deslorelin to control reproduction and/or to prevent or suppress sex hormone-related behavior and disease in male and female cats.^4^

**Figure:**
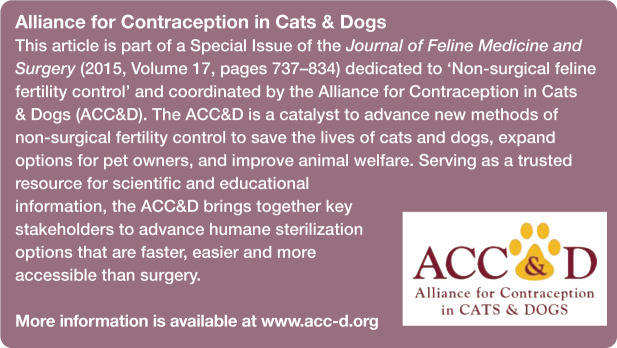


## Physiological feasibility

The ease of administration, efficacy, safety and reversibility of deslorelin implants explain why they are perceived as a promising method for controlling male and female domestic cat reproduction; in particular by the owners of cats temporarily not intended for breeding, but also by veterinary practitioners for cats at increased anesthetic risk and by organizations in areas without easy access to a surgical facility.

In most feline studies published in the scientific literature, deslorelin implants are inserted subcutaneously through a needle between^5–12^ or in the region of the shoulder blades.^13,14^ If easy removal of the implant is desired (eg, in an attempt to shorten the duration of action), the implant may also be inserted into the umbilical area.^15,16^ Anesthesia or sedation are not required.^4,5,11^

The aim of this review is to critically appraise the available data on the off-label use of Suprelorin 4.7 mg and Suprelorin 9.4 mg for the control of reproduction in male and female domestic cats, including both adult and prepubertal populations. Pivotal information used in this review has been obtained from scientific papers in peer-reviewed journals,^5,10–14,17–20^ while supportive information has been sourced from meeting summaries and posters.^6,7,9,15,16,21^

**Figure:**
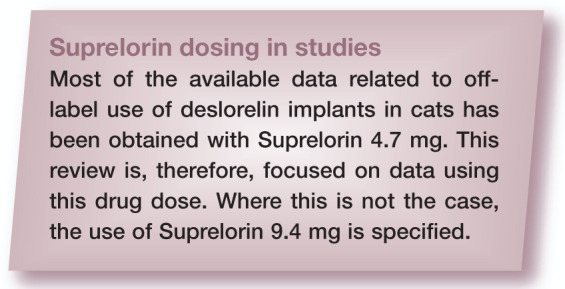


## Evidence base

### Pharmacodynamics of deslorelin implants in cats

In cats, neither the deslorelin release profile nor the LH/FSH patterns after treatment have been characterized. The efficacy of Suprelorin in cats is therefore documented through the use of indirect markers including testosterone, progesterone or estradiol concentrations.^5,11,17,22^

The response to deslorelin, as observed with other GnRH agonists, is biphasic with an initial stimulation period lasting a few days/ weeks followed by a long-lasting suppression period.^23^ Following the stimulation phase post-deslorelin implant insertion, during which increases in progesterone and/or estradiol have been observed in female cats,^11^ all studies have demonstrated an extended period of reproductive suppression in both adult male cats (n = 10, 1–6 years of age)^5^ and adult female cats (n = 20, 2–5 years of age).^11^ During the suppression period, steroid concentrations below 1 ng/ml for progesterone and 10 pg/ml for estradiol have been observed in females;^11^ in male cats, plasma testosterone concentration has remained at basal levels (<0.1 ng/ml).^5^

**Figure 1:**
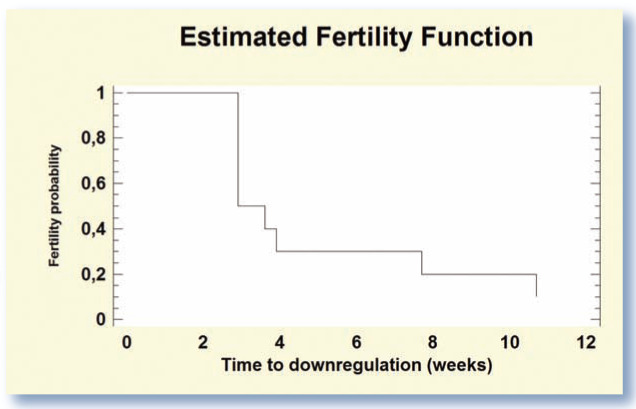
Probability of downregulation (testosterone plasma concentration <0.1 ng/ml) in 10 sexually mature male cats according to the time after treatment with Suprelorin 4.7 mg. *Data extracted from Goericke-Pesch et al*^5^

The time interval between Suprelorin insertion and the drop in steroid concentrations to basal levels is defined in this review as the ‘time to downregulation’. In a study involving 10 adult male cats (1–6 years old), the curve describing time to downregulation, presented in Figure 1, demonstrates that downregulation was reached in 90% (n = 9/10) of the cats after 3 months.^5^ Desensitization was achieved in half of the cats within 20 days of treatment, but other cats needed more time. Reasons for this large variability are not yet understood.

The ‘duration of action’ of Suprelorin can be defined as the interval between desensitization and recurrence of ovarian or testicular function. In female cats, the time to return to continuous seasonal cycling,^11^ as assessed by recurrence of a new estrous period including typical sexual behavior, was 680.4 ± 62.0 days (mean ± SD, n = 19/20), ranging from 483 days (approximately 16 months) to 1025 days (approximately 37 months).^11^ In one female cat, the implant was still active at the end of the study, with a duration of action of more than 1102 days (>37 months).^11^ Noteworthy is the large variability in the duration of action of Suprelorin.

In a study involving seven adult male cats (1–6 years of age), and using plasma testosterone concentration exceeding 0.5 ng/ml as the threshold for resumption of gonadal function, duration of action was 78.8 ± 12.9 weeks (mean ± SD) and ranged from 61.7 weeks (approximately 15 months) to 100.7 weeks (approximately 25 months).^17^

Overall, studies demonstrate a similar duration of efficacy in female and male cats, and also very large individual variability in this parameter in the two sexes. Factors involved in this variability have not been clarified in cats. Some authors suggest an individual susceptibility to deslorelin,^11,13,17^ while the impact of vascularization at the insertion site, of drug metabolism in the individual animal and of individual variation in the desensitization mechanism has not been explored.

### Clinical effects of slow-release deslorelin implants in male cats

Pivotal studies focused on sexually mature male cats assessed the clinical effects of Suprelorin both in experimental conditions, with animals 1–6 years old (n = 10 and n = 7),^5,17^ and in field conditions with privately owned indoor cats 1–4 years old (n = 22 and n = 12).^13,14^ In one additional experimental study of five adult male cats, the age of the animals was not reported.^22^

A significant increase in sexual behavior (libido, mounting and mating) was observed in 8/10 adult male cats^5^ from the time of Suprelorin administration until day 16 after treatment, likely owing to the stimulation phase of the treatment. After stimulation, disappearance of penile spikes^5^ and significant decreases in testicular volume^5^ and in sexual behavior^5^ were observed in 10/10 treated male cats.

In two studies, complete disappearance of penile spikes was recorded 9.4 ± 1.0 weeks (mean ± SD, n = 10)^5^ and 5.8 ± 1.1 weeks (range 4–7 weeks) (mean ± SD, n = 5)^22^ after treatment. In one of these studies, involving 10 cats,^5^ testicular volume (mean volume of right and left testes), relative to pretreatment values, was significantly decreased by approximately 25% at week 4, by about 60% at week 12 (approximately 3 months) and by 73.5% at week 36 (approximately 9 months).^5^ Similar results were observed in another study run in field conditions, with a significant decrease in testicular volume of 29.8% after 1 month (n = 22), 48% at 2 months (n = 18), 54% at 3 months (n = 15) and 60.6% at 4 months (n = 12) post-treatment.^13^

Sexual behavior (libido, mounting and mating) was also assessed in a study involving 10 experimental cats kept in the presence of a female in estrus.^5^ No interest in an estrous female cat was observed commencing from weeks 11 (n = 3), 12 (n = 5) and 16 (n = 2), and this continued until the end of the observation period at 36 weeks (approximately 9 months).^5^

Studies have assessed the impact of Suprelorin on semen quality. Novotny et al showed that sperm counts were significantly decreased compared with pretreatment values, with a median of 0.001 x 10^6^ spermatozoa per ejaculate (range 0.000 x 10^6^ to 18.000 x 10^6^) at week 16 (n = 12).^13^ Another study observed complete azoospermia in all assessed cats (4/4), but not until 6 months after treatment,^14^ indicating that there is a significant delay between the initial changes in sperm production and the onset of infertility. This is to be expected given that the sperm production cycle in tom cats lasts 46.8 days.^24^

Cats treated with deslorelin demonstrated multiple behaviors commonly observed in male cats following surgical castration. Urine marking disappeared in all cats (n = 10) within 10 weeks of treatment.^5^ Furthermore, a small supportive study involving six cats demonstrated a gradual cessation of aggression towards the owner (scratching, biting), and reduction of vocalization and the strong intact male cat smell within 18.7 ± 3.5 days (range 10–35 days).^7^ This lasted for at least 6–8 months, until the end of the observation period.^7^ Finally, in their study involving 10 cats,^5^ Goericke-Pesch et al observed a significant increase in appetite 7.5 months following treatment,^5^ similar to that commonly seen after surgical castration.

Duration and reversibility of long-term deslorelin effects in male cats were assessed in a single study involving seven experimental cats.^17^ Pretreatment testicular volumes were reached 6.9 ± 3.4 weeks (range 5–11 weeks)^17^ (mean ± SD, n = 7) after the last basal testosterone concentration was recorded, on average approximately 21 months after implant administration.^17^ Time of reappearance of penile spikes was idiosyncratic. They reappeared in 2/7 cats while testosterone was still at basal levels; while, in the other cats, normal-size spikes were observed 10.8 ± 2.3 weeks (mean ± SD, n = 5/7) after the last basal testosterone concentration was recorded.^17^ Normal penile spikes were observed in these five cats on average approximately 21 months after treatment.^17^

Return to fertility was assessed in four cats allowed to mate estrous female cats until they became pregnant.^17^ Libido first resumed around 22 months after treatment, and 1–3 additional months were needed for normal mating behavior to occur.^17^ Fertile matings were achieved between 7 and 42 weeks (approximately 10 months) after the last basal testosterone concentration was recorded.^17^ The resulting litter size was 4.0 ± 0.0 kittens.^17^ Although obtained on a very small sample, these results suggest high variability in the time to return to full fertility after long-term downregulation induced by Suprelorin.

Following treatment with a 4.7 mg deslorelin implant, no local reaction (eg, swelling or scratching) was observed.^5,13^ There was no change in the cats’ health status based on clinical examinations, and blood counts and serum biochemistry throughout Suprelorin treatment remained in the normal interval.^5,7,9,15,21^

### Clinical effects of slow-release deslorelin implants in female cats

Three pivotal studies have addressed the clinical effects of Suprelorin in sexually mature female cats.^10–12^ All trials were performed under experimental conditions. Two evaluated Suprelorin 4.7 mg in 10 and 20 adult female cats, respectively.^10,11^ The third study was a controlled trial in which 28 adult female cats were allocated to three groups that received: one Suprelorin 9.4 mg implant alone (n = 14); one Suprelorin 9.4 mg implant together with concomitant megestrol actetate treatment (n = 7); or a placebo implant (n = 7).^12^

During the stimulation period, the increase in steroid concentrations was associated with a behavioral estrus in 2/20^11^ and 4/10^10^ female cats after treatment with a Suprelorin 4.7 mg implant. Typical estrous behavior started by 3.8 ± 2.2 days (mean ± SD) post-treatment^10^ and lasted for 3.5 ± 3.1 days^10^ (n = 4/10). Following the 9.4 mg deslorelin treatment, estrous behavior was observed 2 days after treatment in 2/14 female cats belonging to the Suprelorin-only group.^12^

Despite estrus-like fecal estradiol concentrations, no estrous behavior was observed during the stimulation period in 7/7 female cats treated with 5 mg oral megestrol acetate at 14 days before, 12 h before and 14 days after administration of a 9.4 mg deslorelin implant.^12^ These results are encouraging, but the small study size precludes any firm conclusion as to whether megestrol acetate administration prior to Suprelorin treatment is an efficient strategy to prevent induced estrus.

The time to return to continuous seasonal cycling was assessed in a single study involving 20 female cats that received Suprelorin 4.7 mg.^11^ The first post-treatment estrous period appeared in 680.4 ± 62.0 days (mean ± SD, n = 19/20) (range 483 days [approximately 16 months] to 1025 days [approximately 37 months]).^11^ The high variability in this parameter mirrors that observed in male cats.

Resumption of full fertility was assessed in eight female cats mated after the end of efficacy of Suprelorin 4.7 mg.^11^ Seven female cats became pregnant immediately. All eight females delivered naturally and spontaneously. Litter size was 3.3 ± 1.5 kittens (mean ± SD), ranging from one to five kittens.^11^

Both Suprelorin implants (4.7 mg and 9.4 mg) were well tolerated by adult female cats in the aforementioned pivotal studies.^10–12^ Occasionally, slight and temporary adverse reactions at implant sites in the subcutaneous interscapular area were observed, including swelling of the skin (n = 1/20),^11^ pyodermatitis (n = 1/10),^10^ edema (n = 3/21)^12^ and an erosive laceration due to scratching (n = 1/21).^12^

In a single case report, a female cat unintentionally treated with Suprelorin 4.7 mg 8–9 days after mismating delivered four healthy kittens 66 days after the mismating, but showed no interest in the kittens and milk production was inadequate.^20^ Reproductive behavior and parameters were reportedly normal at the subsequent pregnancy.^20^

To assess the ability of Suprelorin to postpone puberty, one pivotal trial involved 30 prepubertal female cats treated when 114.4 ± 12.7 (mean ± SEM) days (approximately 4 months) old and weighing 1.5 ± 0.1 kg (mean ± SEM).^19^ Fourteen of the 15 treated kittens reached puberty (diagnosed by estrous behavior and vaginal cytology) when 281.2 ± 21.6 days old (mean ± SEM) (range 180–428 days).^19^ This was significantly later than the 15 queens of the control group, which displayed puberty when 177.8 ± 10.8 days old (mean ± SEM) (134–286 days).^19^ Body weight at puberty was not different between the treated and the control groups.^19^ However, one treated queen showed induced estrus and another showed clinical signs of pyometra 13 and 92 days, respectively, after treatment.^19^

## Challenges

Despite the fact that prevention of gonadal function and fertility by deslorelin implants has been shown to be safe, reversible and effective at managing undesirable sexual behavior, this GnRH agonist is currently not approved by any regulatory agencies for use in cats. Hence all use in cats is off-label.

There are two main challenges that need to be addressed before Suprelorin may be successfully used in cats. Firstly, owing to the large variability in the duration of efficacy, it is very difficult to define the duration of effect for the label claim – and, therefore, the time for retreatment if an owner wishes to continue contraception. Secondly, the stimulation phase during the first weeks after implant administration is associated in some female cats with a fertile estrus that needs to be carefully managed to avoid unwanted pregnancies. The efficacy of the two possible methods for reducing the occurrence of induced heats (progestogens and treatment of prepubertal queens) needs to be carefully evaluated.

Additionally, studies addressing the impact of deslorelin on growth (in particular on the time of epiphyseal closure), and on the phenotype of treated prepubertal cats, are lacking.

## Conclusion

It is concluded that, although off-label, Suprelorin appears to be a safe, convenient and efficient reversible contraceptive for male and female cats.

## Key Points

- Deslorelin implants have been shown to effectively, safely and reversibly postpone puberty or suppress reproductive function and related behaviors in male and female cats.
- In sexually mature cats, the duration of efficacy of a 4.7 mg deslorelin implant has been shown to be approximately 20 months (range 15–25 months) in males and approximately 24 months (range 16–37 months) in females.
- The effects of deslorelin are reversible, as demonstrated by fertile matings approximately 2 years post-treatment in both male and female adult cats.
- However, the high variability in the onset of downregulation, duration of efficacy and return to full fertility are potential challenges for practitioners and owners.
- Deslorelin implants are registered and sold in select countries under the brand name of Suprelorin for long-term contraception in adult male dogs and ferrets.
- The market need for a long-term contraceptive method for cats, combined with the encouraging preliminary results obtained with deslorelin, should encourage the development of a new cat indication for Suprelorin implants once these initial results have been confirmed in large clinical studies.

**Figure:**